# Pathophysiological Significance of GM3 Ganglioside Molecular Species With a Particular Attention to the Metabolic Syndrome Focusing on Toll-Like Receptor 4 Binding

**DOI:** 10.3389/fmolb.2022.918346

**Published:** 2022-05-30

**Authors:** Jin-ichi Inokuchi, Hirotaka Kanoh

**Affiliations:** ^1^ Division of Glycopathology, Institute of Molecular Biomembranes and Glycobiology, Tohoku Medical and Pharmaceutical University, Sendai, Japan; ^2^ Forefront Research Center, Graduate School of Science, Osaka University, Osaka, Japan

**Keywords:** GM3 ganglioside, innate immunity, Toll-Like Receptor 4, Inflammation, metabolic syndrome

## Abstract

GM3 ganglioside, the first molecule in ganglioside family biosynthesis, is formed by transfer of sialic acid to lactosylceramide. Several dozen GM3 molecular species exist, based on diversity of ceramide structures. Among ceramide structures composed of sphingosine and fatty acids, there is a great diversity resulting from different combinations of chain length, hydroxylation, and unsaturation of fatty acid chains. Expression patterns of GM3 species in serum vary during pathogenesis of metabolic syndrome. Physiological activity of each species, and significance of the variability, are poorly understood. Our studies revealed that GM3 species with differing fatty acid structures act as pro- or anti-inflammatory endogenous Toll-like receptor 4 (TLR4) ligands. Very long-chain fatty acid (VLCFA) and α-hydroxyl VLCFA GM3 variants strongly enhanced TLR4 activation. In contrast, long-chain fatty acid (LCFA) and ω-9 unsaturated VLCFA GM3 variants suppressed TLR4 activation. GM3 interacted with extracellular TLR4/myeloid differentiation factor 2 (MD-2) complex, thereby promoting dimerization/oligomerization. In obesity and metabolic syndrome, VLCFA-variant GM3 species were elevated in serum and adipose tissue, whereas LCFA-variant species were reduced, and such imbalances were correlated with disease progression. Our findings summarized in this review demonstrate that GM3 molecular species are disease-related endogenous TLR4 ligands and modulate homeostatic and pathogenic innate immune responses.

## 1 Introduction

Innate immune responses (host defenses against pathogens) are a major contributing factor in physiological homeostasis. On the other hand, chronic persistence of innate immune responses (*i.e.*, chronic inflammation) may lead to development of a variety of serious diseases, including malignant tumors and metabolic syndrome (Lumeng and Saltiel, 2011; Hotamisligil, 2017). Elucidation of the molecular mechanisms whereby innate immune responses as a homeostatic mechanism are transformed into chronic inflammatory responses that lead to pathogenesis will be useful in formulation of novel diagnostic and therapeutic methods.

This review is focused on molecular mechanisms underlying the role of glycosphingolipids (GSLs) in onset and progression of metabolic syndrome, a group of five conditions that often lead to heart disease, diabetes, and/or stroke. In particular, we summarize the role of GM3 ganglioside and its fatty acid (acyl) chain structure in regulation of innate immune responses, including our own recent findings.

## 2 Molecular Basis of Toll-Like Receptor 4-Mediated Innate Immune Responses

Chronic inflammation in metabolic syndrome is apparently caused by activation of pattern recognition receptors such as Toll-like receptors (TLRs) and C-type lectin receptors, and of downstream transcription factor NF-κB (Baker et al., 2011; Moresco et al., 2011; Kawai and Akira, 2011; Tanaka et al., 2014). A complex of TLR4 and co-receptors [myeloid differentiation factor 2 (MD-2) and cluster of differentiation 14 (CD14) molecules] recognizes exogenous pathogen-associated molecular patterns (PAMPs); *e.g.*, lipopolysaccharide (LPS) as a ligand (Kawai and Akira, 2011; Moresco et al., 2011). LPS is a glycolipid usually derived from outer cell wall membrane of infectious Gram-negative bacteria; it is also termed “endotoxin” because of its proinflammatory activity. Elevated total endotoxin levels in sera of obese and metabolic syndrome patients have been reported, although the cause and measurement methods of these observations remain controversial (Cani et al., 2007). High-mobility group box one protein (HMGB1), which functions primarily as a nuclear protein, is released from chromosomes of dead cells and from hypertrophied adipocytes in metabolic syndrome patients, and functions secondarily as an endogenous ligand for TLR4 (Harris et al., 2012; Guzman-Ruiz et al., 2014). Free fatty acids released from hypertrophic adipocytes and from fetuin-A, which functions as a carrier protein, are similarly involved in TLR4 activation in metabolic syndrome (Shi et al., 2006; Pal et al., 2012). Cold-inducible RNA-binding protein (CIRP), which is released by cold stimuli, and serum amyloid A (SAA), which is involved in TLR4-mediated cancer metastasis, also trigger TLR4 activation (Hiratsuka et al., 2008; Qiang et al., 2013). These endogenous ligands are collectively referred to as damage-associated molecular patterns (DAMPs) derived from cellular or tissue abnormalities, and sometimes as danger signals or alarmins.

TLR4-KO mice showed reduced metabolic syndrome symptoms, *e.g.*, abnormal glucose metabolism (Shi et al., 2006), suggesting that TLR4 activation by various exogenous and endogenous ligands is an important contributor to the pathogenic processes.

## 3 Regulatory Mechanisms of Innate Immune Responses Mediated by Sphingolipids

Research on activation and regulation mechanisms of innate immune responses mediated by sphingolipids has progressed rapidly during the past decade. Glucosylceramide (GlcCer), the molecule produced by addition of glucose to ceramide, activates Mincle (macrophage-inducible C-type lectin), a C-type lectin receptor in dendritic cells (Nagata et al., 2017). Mincle expression is upregulated in adipose tissue of obese mice, and metabolic syndrome symptoms were reduced in Mincle-KO mice (Ichioka et al., 2011; Tanaka et al., 2014). Lactosylceramide (LacCer), produced by addition of galactose to GlcCer, is involved in recognition of the glycolipid lipoarabinomannan in mycobacterial cell walls by neutrophils, and promotes (through signal transduction) maturation of phagocytic cells and activation of bactericidal mechanisms following phagocytosis (Nakayama et al., 2016). Globo-series sphingolipids Gb3 (produced by addition of galactose to LacCer) and Gb4 (produced by addition of N-acetylgalactosamine to Gb3) are involved in regulation of TLR4 activation in macrophages and vascular endothelial cells (Kondo et al., 2013; Nitta et al., 2019).

**FIGURE 1:**
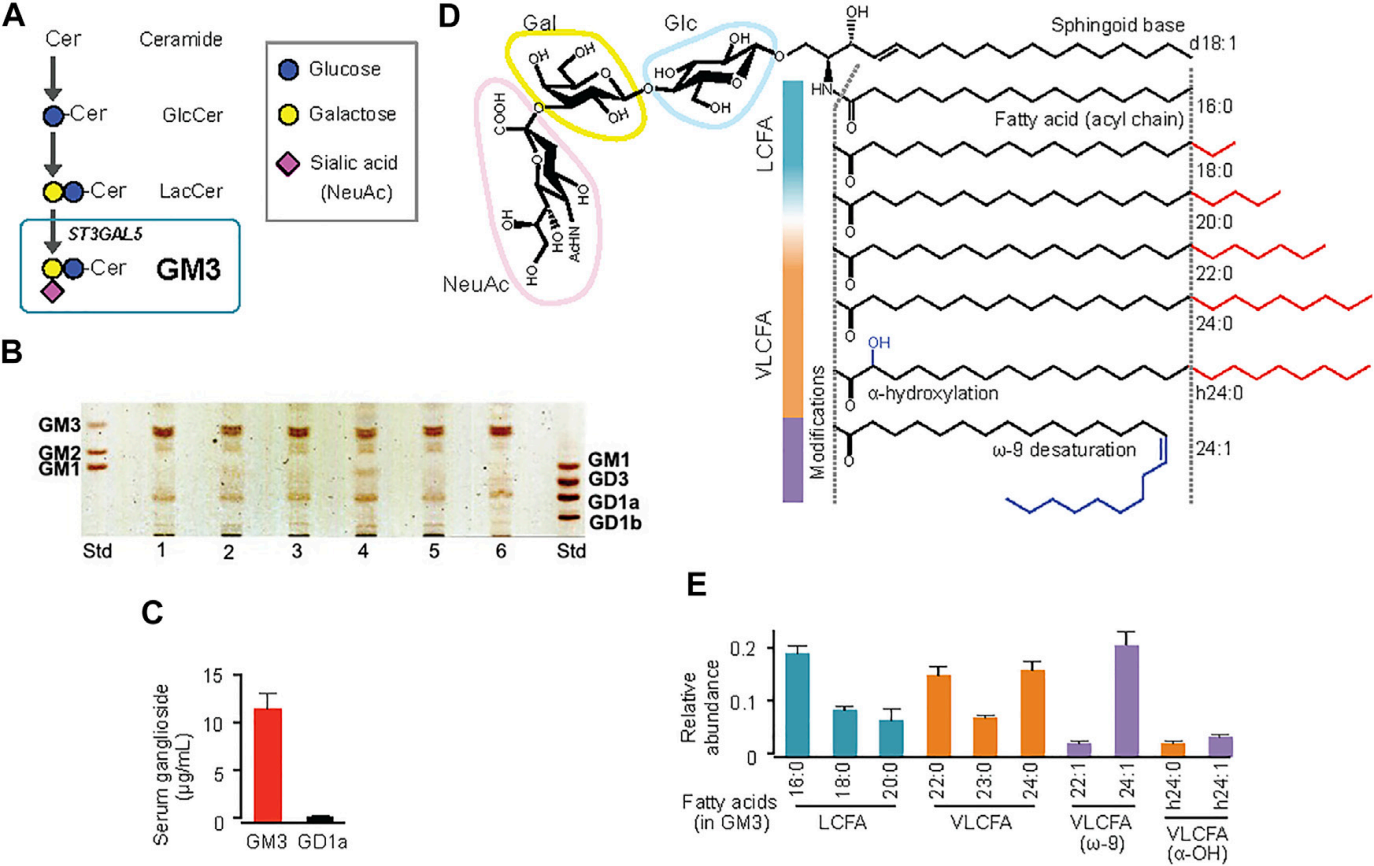
Molecular species of ganglioside GM3 in human serum, and their acyl-chain structures. **(A)** Pathway (schematic) of GM3 biosynthesis from ceramide. **(B)** TLC analysis of ganglioside species in human serum. **(C)** Quantification by densitometry of major ganglioside species GM3 and GD1a in sera from healthy subjects (*n* = 6). **(D)** Detailed structures of GM3 species: sialyllactose head group, sphingoid base (d18:1), typical fatty-acid lengths (LCFA, VLCFA), and acyl-chain modifications (a-hydroxylation, ω-9 unsaturation). **(E)** Quantification by LC-MS/MS of serum GM3 species in healthy subjects. Total abundance of 10 representative species was defined as 1. (Kanoh et al., 2020).

**FIGURE 2:**
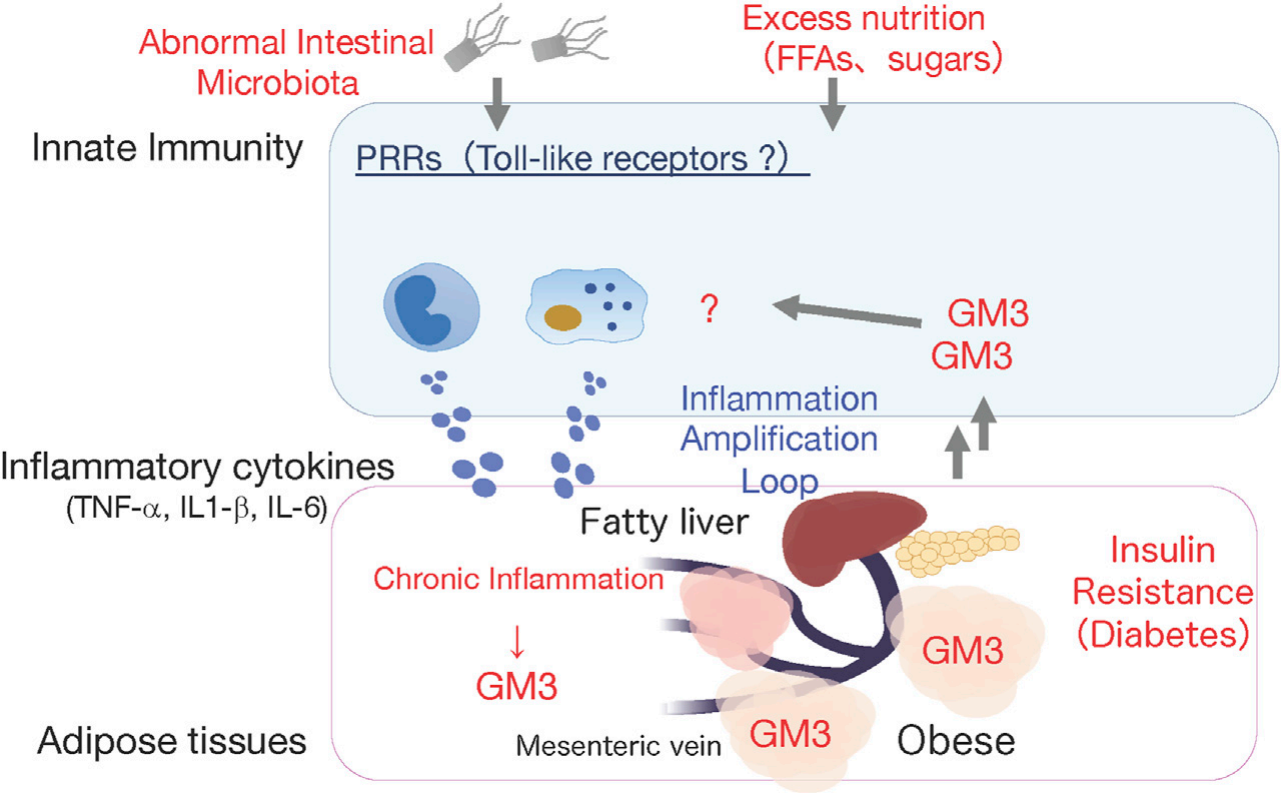
Working hypothesis: GM3 is involved in activation of innate immune responses. High fat diet-induced obese GM3S-KO mice showed reduced obesity-induced systemic insulin resistance, and absence of chronic inflammation in visceral adipose tissues (Nagafuku et al.). This observation led us to hypothesize that the increased GM3 might activate innate immune responses producing inflammatory cytokines.

Ganglioside GM3, produced by addition of sialic acid to LacCer (Figure 1A), is expressed mainly in adipose tissue and muscle in humans and mice, and liver and serum in humans (Senn et al., 1989; Wentworth et al., 2016; Go et al., 2017; Inokuchi et al., 2018). GM3 expression in adipocytes is induced by stimulation of the inflammatory cytokines TNF-α and IL-1β, derived from tissue macrophages (Tagami et al., 2002; Nagafuku et al., 2015). In obesity, adipose tissue is infiltrated by macrophages, and chronic inflammation caused by inflammatory cytokine production leads to insulin resistance (Lumeng and Saltiel, 2011; Hotamisligil, 2017). GM3 expression in visceral adipose tissue, and gene expression of GM3 synthase (GM3S; St3gal5), were significantly elevated in *ob/ob* mice (which display obesity and metabolic syndrome because of deficiency of the appetite suppressor hormone leptin) and in mouse models of obesity induced by high-fat diet (Tagami et al., 2002; Nagafuku et al., 2015). Molecular imaging of living cells suggests that increased GM3 levels promote insulin resistance by increasing the rate of insulin receptor spreading from caveola-microdomain (lipid rafts) and decreasing signaling efficiency (Kabayama et al., 2007). Conversely, inhibition of GM3 synthesis by GlcCer synthase inhibitors (D-PDMP, Genz-123346) enhanced insulin signaling in adipocytes (Tagami et al., 2002; Zhao et al., 2007). GM3S-KO mice showed increased systemic insulin sensitivity and reduction of obesity-induced chronic inflammation (Yamashita et al., 2003; Nagafuku et al., 2015). These findings suggest the existence of a GM3-mediated chronic inflammatory mechanism upstream of insulin resistance, and involvement of GM3 in innate immune responses (Figure 2).

## 4 Regulatory Mechanism of Toll-Like Receptor 4 Activation *Via* Fatty Acid Structure of Ganglioside Gm3 Molecular Species

**FIGURE 3:**
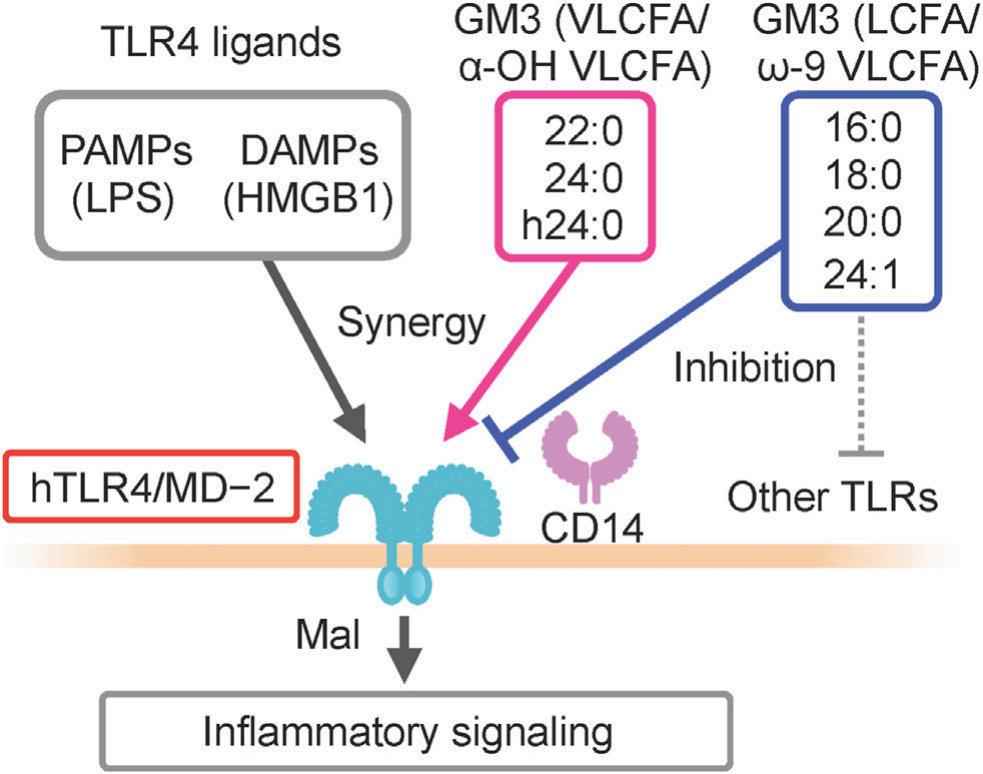
Pro- and anti-inflammatory GM3 molecular species against human TLR4/MD2 complex.

GM3 is the predominant ganglioside component of human sera, with concentrations in the 10–15 μg mL^−1^ (∼10 µM) range (Figure 1). Combinations of ceramide structures in GM3 are highly diverse, resulting in many GM3 molecular species. In particular, the fatty acid chains (acyl chains) vary in length because they are composed of many possible long-chain fatty acid (LCFA) [16:0, 18:0, 20:0] and very long-chain fatty acid (VLCFA) [22:0, 23:0, 24:0] structures, and can be structurally modified by α-hydroxylation and ω-9 unsaturation (Figures 1D,E). Serum expression patterns of GM3 molecular species fluctuate during pathogenesis of metabolic syndrome (Veillon et al., 2015); however, the significance of such fluctuations, and the bioactivities of particular molecular species, are poorly understood. We examined physiological activities of representative GM3 molecular species (16:0, 18:0, 20:0, 22:0, 24:0, h24:0, 24:1) using innate immune responses as an indicator (Kanoh et al., 2020), and made the following observations: 1) LCFA variants (16:0, 18:0) suppressed proinflammatory cytokine production mediated by human TLR4/MD-2 complex, whereas VLCFA variants (22:0, 24:0, h24:0) strongly promoted TLR4 activation. 2) Among VLCFA variants, the unsaturated one (24:1) had an inhibitory effect on TLR4. 3) GM3 species alone did not alter proinflammatory cytokine production; they showed distinctive effects as above only in the presence of TLR4 ligands (LPS, Lipid-A, HMGB1). 4) These effects were selective for activation of TLR4 but not of other TLR family members (TLR1/2 by Pam3-CSK4, TLR2/6 by MALP-2, TLR5 by flagellin, TLR7/8 by R848). Thus, GM3 molecular species are evidently TLR4-selective endogenous ligands that display either pro- or anti-inflammatory properties depending on their fatty acid structures (Figure 3). Studies by K. Furukawa’s group and ours suggested that globo-sphingolipids modulate activation of TLR4/MD-2, and that VLCFA-variant Gb3 species mediate chronic inflammation in diabetic nephropathy (Kondo et al., 2013; Nitta et al., 2019). The above findings, taken together, indicate that regulation by certain GSLs of innate immune responses based on fatty acid chain length is selective for TLR4 and its surrounding regulators.

We also examined bioactivity of GM3 molecular species in innate immune responses mediated by mouse TLR4/MD-2 complex. VLCFA-variant GM3 species strongly promoted TLR4 activation, as in humans. On the other hand, LCFA-variant and unsaturated fatty acid-variant GM3 species weakly promoted TLR4 activation, in contrast to the inhibitory effect observed in humans. Thus, all GM3 species seem to have proinflammatory effects on mouse TLR4. Why does the differential selectivity of GM3 species between human and mouse occur and how is it related to the GM3 recognition mechanism by TLR4/MD-2?

## 5 Comparative Fatty Acid Structure/Activity Relationships in Lipopolysaccharide and Ganglioside Molecular Species

**FIGURE 4:**
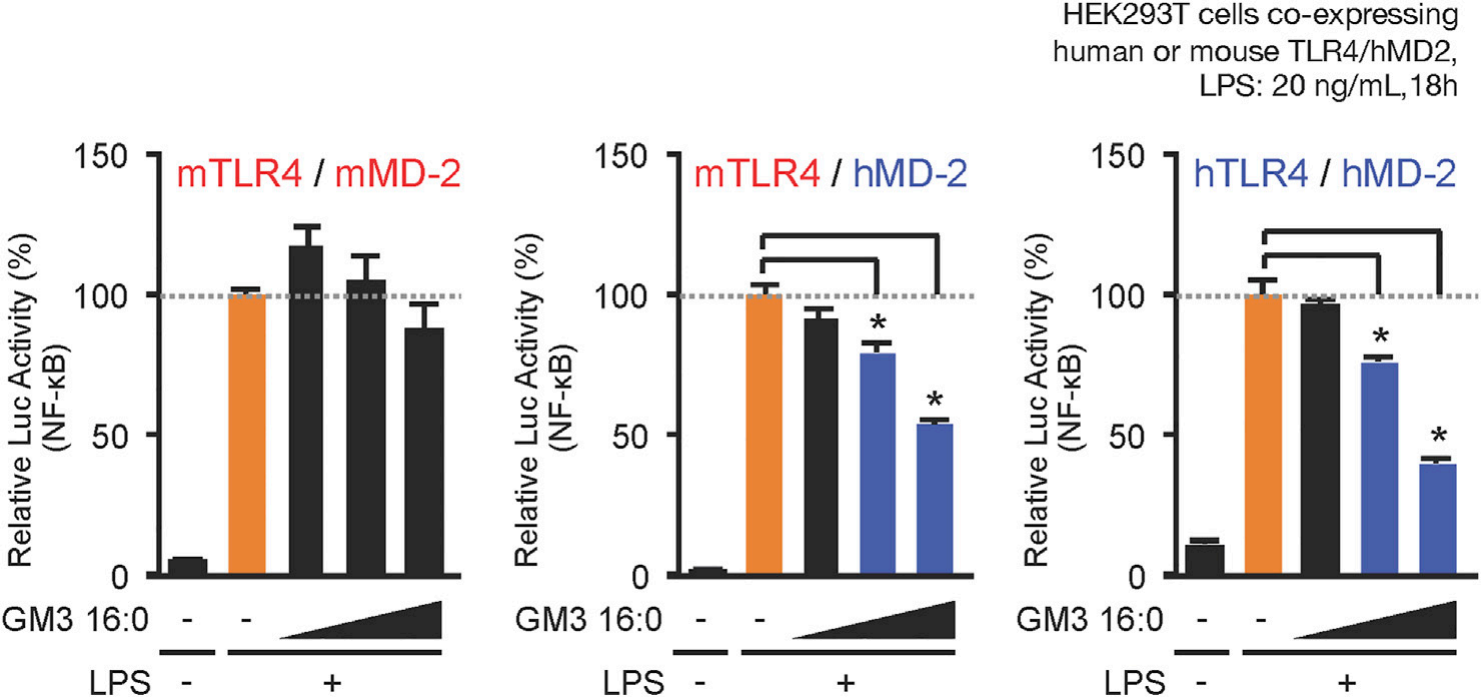
MD-2 (hydrophobic pocket) is the basis for selectivity for acyl chains of GM3 species. Comparative inhibitory effects of GM3 16:0 on LPS-induced activation of mouse mTLR4/mMD-2 complex, human hTLR4/hMD-2 complex, and mTLR4/hMD-2 chimeric complex. Signal transduction was monitored by NF-kB reporter assay (Kanoh et al., 2020).

**FIGURE 5:**
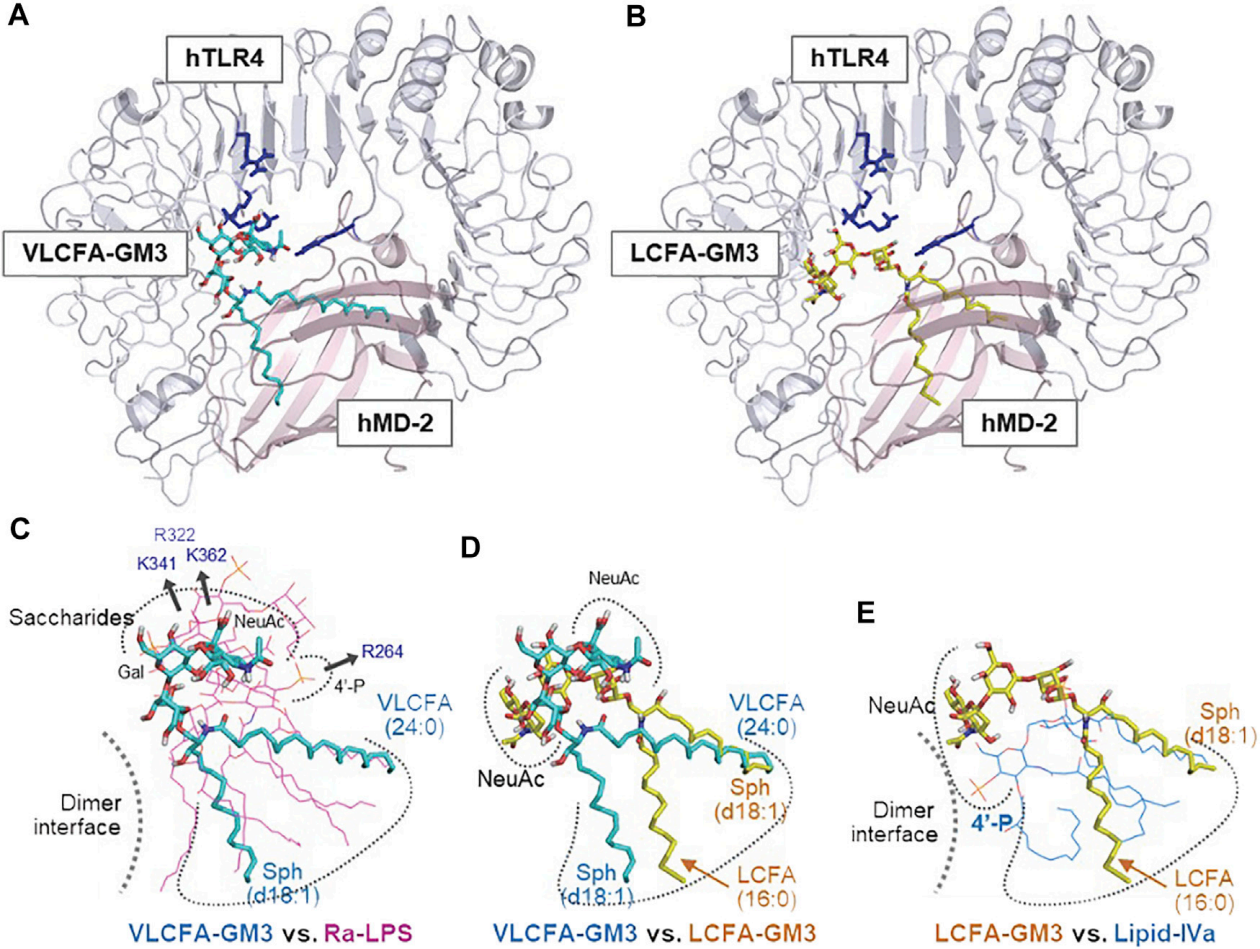
Enhancement or suppression of human TLR4 activation by GM3 species. Ligand-macromolecular docking analysis: Docking model of GM3 24:0 **(A)** and 16:0 **(B)** binding to human TLR4/MD2 complex. Basic residues of TLR4 are colered in blue. Superposition of GM3 24:0 vs. Ra-LPS **(C)**, GM3 24:0 vs. GM3 16:0 **(D)**, and GM3 16:0 vs. lipid IVa **(E)**. Binding mode of lipid IVa (antagonist) differed from that of LPS; it involved reverse orientation of 4′-phosphate and acyl chains, which may inhibit TLR4/MD-2 dimer formation **(E)**. Binding mode of GM3 16:0 was opposite that of GM3 24:0; it involved reverse orientation of acyl chains to MD-2 hydrophobic pockets **(D)**, resulting in suppression of TLR4 activation **(E)** (Kanoh et al., 2020).

When LPS acts as a TLR4 ligand, its glycan structure is recognized by TLR4 and its fatty acid structure is recognized by MD-2 (Park et al., 2009; Ohto et al., 2012). GM3, like LPS, has glycan chains consisting of glucose, galactose, and sialic acid and a ceramide moiety containing a variety of fatty acid structures. MD-2 may therefore be involved in recognition of GM3 fatty acid structures. We compared physiological activities of GM3 16:0 in mouse TLR4/MD-2 complex, human TLR4/MD-2 complex, and a chimeric mouse TLR4/human MD-2 complex. GM3 16:0 displayed inhibitory effects on the latter two complexes (Figure 4); *i.e.*, GM3 bioactivity based on fatty acid structure was dependent on MD-2. These findings suggest that GM3 regulates TLR4 in lipid membrane, and exerts its effect on TLR4 *via* MD-2 as a ligand, similarly to LPS (Galanos et al., 1984; Galanos et al., 1985; Wang et al., 1990; Akashi et al., 2001; Mueller et al., 2004; Saitoh et al., 2004). Lipid-A, the core structure of LPS, has six fatty acids and acts as an agonist of both human and mouse TLR4/MD-2. Lipid-IVa, the precursor of Lipid-A, has four fatty acids and acts as an antagonist of human TLR4/MD-2 and a partial agonist of mouse TLR4/MD-2. The correlation between bioactivity and fatty acid number for MD-2 is species-dependent; Lipid-IVa has an inhibitory effect on both mouse TLR4/human MD-2 chimeric complex and human TLR4/MD-2 complex. The Lipid-IVa analog eritoran, a TLR4 inhibitor, contains an unsaturated fatty acid (18:1, ω7) whose binding to MD-2 mediates the inhibitory effect. The unsaturated fatty acid chain at the double bond site binds to the hydrophobic pocket of MD-2 while flipping 180°, thereby reducing apparent chain length and simultaneously increasing binding force (hydrophobicity) (Kim et al., 2007). The relationship for GM3 molecular species between bioactivity and fatty acid chain length and modification, and the dependency on MD-2, are similar to findings for LPS and eritoran, suggesting that the mechanism for regulation of TLR4 activation based on fatty acid chain length is conserved among glycolipid ligands (Figure 5).

## 6 Regulatory Mechanisms of Changes in Fatty Acid Chain Length and Structural Modification of Ganglioside Molecular Species

**FIGURE 6:**
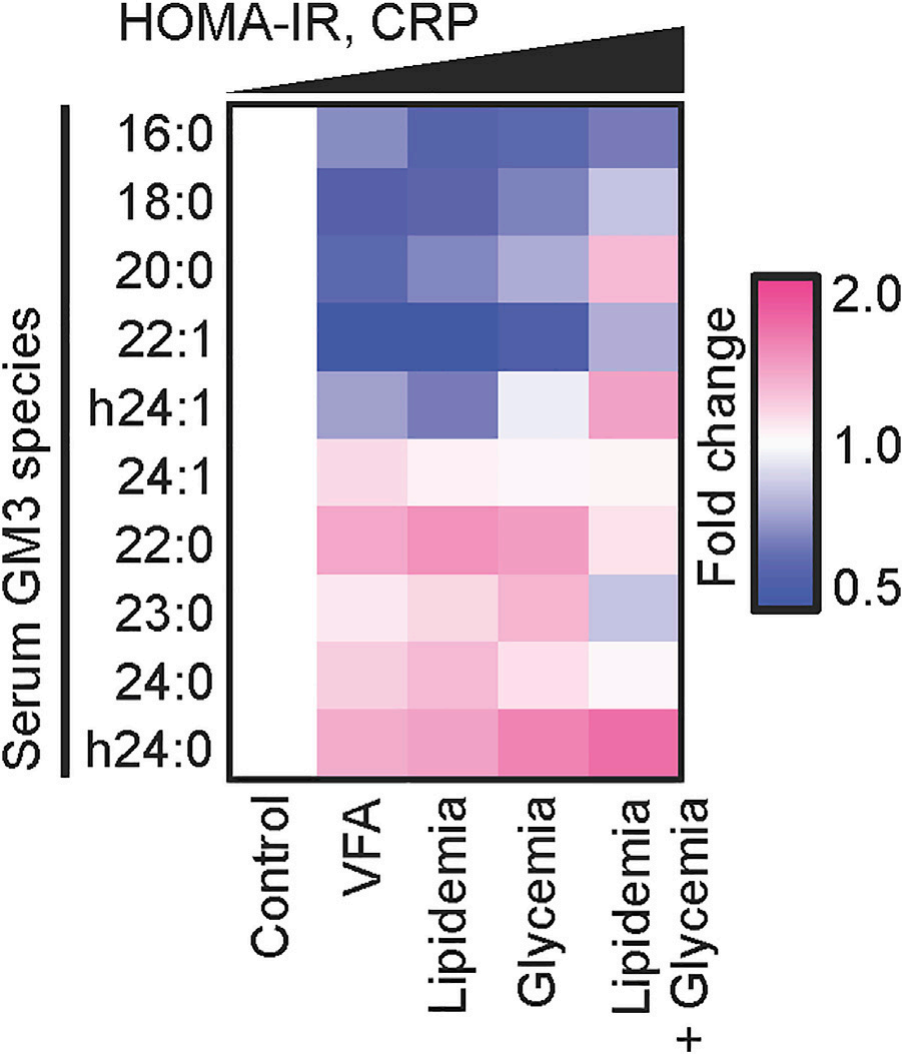
Disease progression and chronic inflammation involve alterations of GM3 species composition. Heat map analysis of serum GM3 species in various pathological phases: control (*n* = 24), VFA (*n* = 38), lipidemia (*n* = 28), glycemia (*n* = 15), lipidemia + glycemia (*n* = 17). Colors (key at right) indicate fold change average of each species relative to control (defined as 1). Order of pathological phases corresponds to increments of homeostasis model assessment for insulin resistance (HOMA-IR) and serum CRP values (Kanoh et al., 2020)

What is the relationship between variations in fatty acid structure of GM3 molecular species as above, and onset/progression of metabolic syndrome? We addressed this question by classifying GM3 species based on their physiological functions in innate immune responses, and by mass spectrometric analysis of expression patterns of GM3 species in sera of metabolic syndrome patients (Kanoh et al., 2020) (Figure 6). Levels of anti-inflammatory GM3 species (16:0, 18:0) were low in unaffected obesity [visceral fat area (VFA) pre-symptomatic phase] and early metabolic syndrome, whereas levels of proinflammatory species (22:0, 23:0, 24:0, h24:0) were much higher. In particular, hydroxylated VLCFA-variant GM3 h24:0 was strongly positively correlated with body mass index (BMI; marker of obesity), abdominal circumference, and C-reactive protein (CRP; marker of chronic inflammation and surrogate marker for inflammatory cytokine IL-6) level. In more severe obesity and metabolic syndrome, expression of VLCFA GM3 declined, while expression of unsaturated-VLCFA GM3 (22:1, 24:1, h24:1) increased. Thus, a proinflammatory shift of GM3 species is evidently associated with obesity and chronic inflammation in early disease stages, while a mechanism that suppresses GM3 proinflammatory properties *via* unsaturation may become operational in advanced (severe) stages. In a mouse obesity model, among GM3 species in visceral adipose tissue, level of hydroxylated-VLCFA GM3 (h24:0) was greatly increased (Kanoh et al., 2020). In humans, elevated hydroxylated-VLCFA GM3 in serum may similarly reflect changes in GM3 species in visceral adipose tissue. TLR4 loss-of-function mutant C3H/HeJ mice showed less increase of GM3 species in visceral adipose tissue (Kanoh et al., 2020). Thus, it appears that proinflammatory GM3 expression is partially dependent on production of proinflammatory cytokines *via* their receptor, TLR4, and that a “proinflammatory loop” consisting of GM3 species and TLR4 is formed, similarly to the case of free fatty acids and TLR4 (Suganami et al., 2007). H. Shimano’s group showed that fatty acid elongase ELOVL is involved in regulation of fatty acid chain length, particularly in Elovl6-KO mice, which have restricted progression of obesity-induced metabolic syndrome (Matsuzaka et al., 2007). Content of C22-C24 fatty acids is lower in these KO mice than in wild-type. Fatty acid unsaturation occurs in late-stage inflammatory responses and is essential for termination of innate immune responses (Oishi et al., 2017). Along this line, unsaturated GM3 content is elevated during severe-phase inflammatory responses. Hydroxylation modification, on the other hand, may be related to modulation of GM3 amount secreted into serum by increased water solubility resulting from hydroxylation, and to enhanced degradation of VLCFAs *via* the α-oxidation pathway (Hama, 2010). Altered composition of GM3 species in obesity may be associated with altered expression of ceramide synthase CerS2/6 and impairment of β-oxidation pathway (Raichur et al., 2014; Turpin et al., 2014). Elucidation of such molecular mechanisms is the focus of ongoing studies.

## 7 Concluding Remarks

Regulatory mechanisms of innate immune responses based on fatty acid chains of sphingolipids are summarized in this review, with focus on ganglioside GM3 and TLR4, and pathogenesis of obesity and metabolic syndrome. TLR4-mediated chronic inflammation plays key roles in pathogenesis of numerous inflammatory diseases and malignancies, and of systemic inflammatory response syndrome (SIRS) in sepsis. High-throughput mass spectrometric techniques will help clarify fluctuating expression patterns of circulating GM3 molecular species in serum, and association of such patterns with many disease processes. Numerous sphingolipids in addition to GM3 are generated from ceramide, and regulatory roles of many of them in innate immune responses are the subject of our ongoing studies.
